# Supplementary figures and images for: Phytobiomes are compositionally nested from the ground up
Source: PeerJ. 2019 Mar 18;7:e6609. doi: 10.7717/peerj.6609 (PMC6428039; doi:10.7717/peerj.6609)

# Dispersion of Beta Diversity

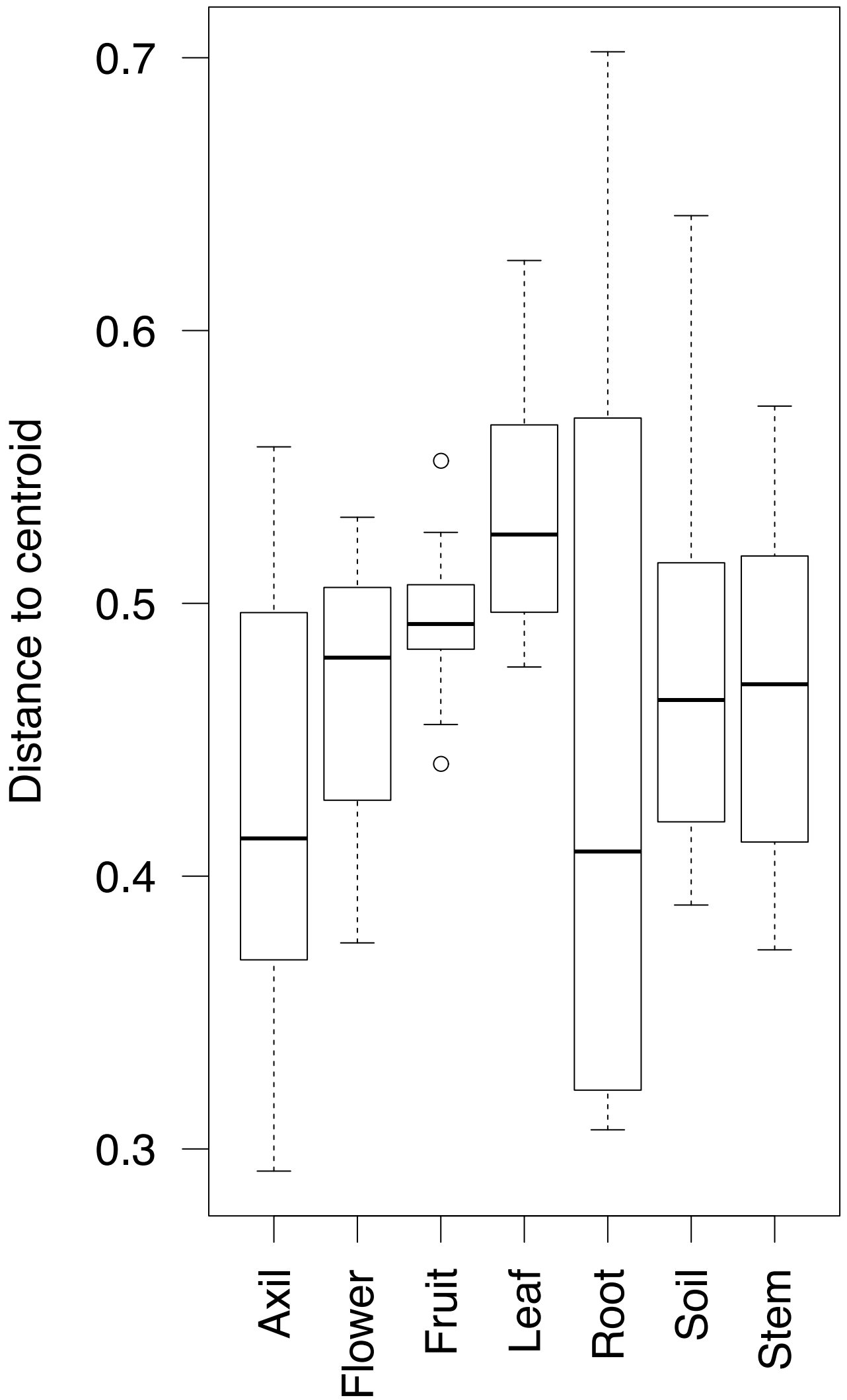

Supplement: Figure S1 — No dispersion means significantly differed in pairwise comparisons. [file peerj-07-6609-s001.pdf]

**Rhizosphere**

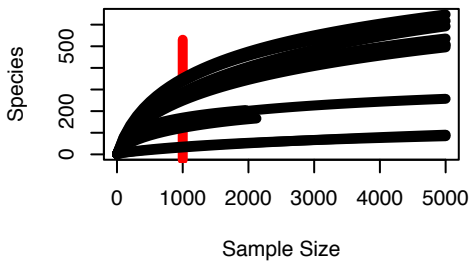

**Soil**

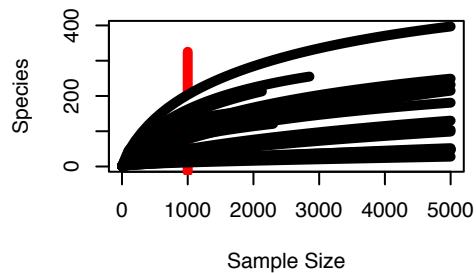

**Flower**

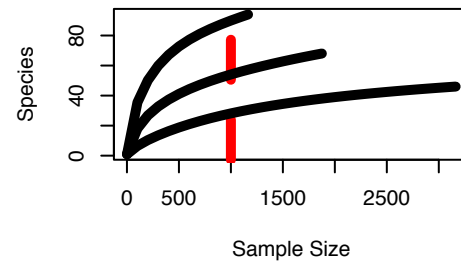

**Axil**

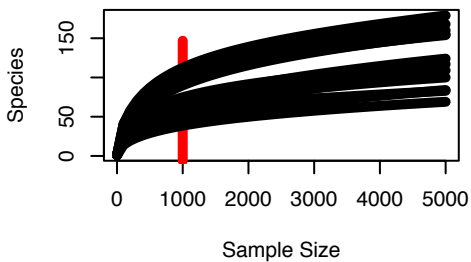

**Stem**

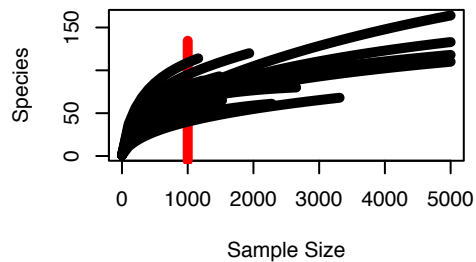

**Leaf**

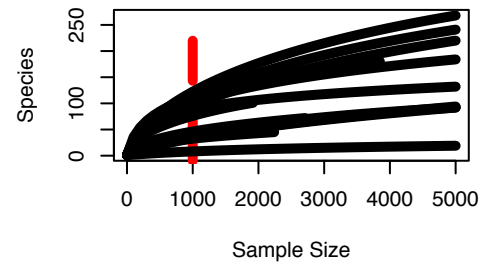

**Fruit**

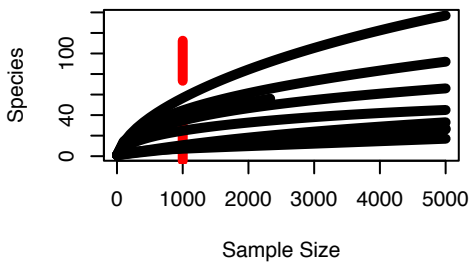

Supplement: Figure S2 — Observed OTU richness is plotted as a function of sequencing depth for each sample. Red vertical line indicates 1000 sequences, the depth at which subsampling occurred. [file peerj-07-6609-s002.pdf]
